# Silent loss and the clinical encounter: Parents’ and physicians’ experiences of stillbirth–a qualitative analysis

**DOI:** 10.1186/1471-2393-12-137

**Published:** 2012-11-27

**Authors:** Maureen C Kelley, Susan B Trinidad

**Affiliations:** 1 Department of Pediatrics, University of Washington School of Medicine, Seattle, USA; 2 Global Alliance to Prevent Prematurity and Stillbirth, Seattle Children’s Hospital, Seattle, USA; 3 Treuman Katz Center for Pediatric Bioethics, Seattle Children’s Research Institute, 1900 9th Avenue – C9 S6, Seattle, WA, 98101, USA; 4 Department of Bioethics and Humanities, University of Washington School of Medicine, 1959 NE Pacific, Box 357120, Seattle, WA, 98195-7120, USA

**Keywords:** Stillbirth, Perinatal death, Perinatal bereavement, Parental support

## Abstract

**Background:**

In the United States, an estimated 70 stillbirths occur each day, on average 25,000 each year. Research into the prevalence and causes of stillbirth is ongoing, but meanwhile, many parents suffer this devastating loss, largely in silence, due to persistent stigma and taboo; and many health providers report feeling ill equipped to support grieving parents. Interventions to address bereavement after neonatal death are increasingly common in U.S. hospitals, and there is growing data on the nature of parent bereavement after a stillbirth. However, further research is needed to evaluate supportive interventions and to investigate the parent-clinician encounter during hospitalization following a stillbirth. Qualitative inquiry offers opportunities to better understand the lived experience of parents against the backdrop of clinicians’ beliefs, intentions, and well-meaning efforts to support grieving parents.

**Methods:**

We present a secondary qualitative analysis of transcript data from 3 semi-structured focus groups conducted with parents who had experienced a stillbirth and delivered in a hospital, and 2 focus groups with obstetrician-gynecologists. Participants were drawn from the greater Seattle region in Washington State. We examine parents’ and physicians’ experiences and beliefs surrounding stillbirth during the clinical encounter using iterative discourse analysis.

**Results:**

Women reported that the cheery, bustling environment of the labor and delivery setting was a painful place for parents who had had a stillbirth, and that the well-meaning attempts of physicians to offer comfort often had the opposite effect. Parents also reported that their grief is deeply felt but not socially recognized. While physicians recognized patients’ grief, they did not grasp its depth or duration. Physicians viewed stillbirth as an unexpected clinical tragedy, though several considered stillbirth less traumatic than the death of a neonate. In the months and years following a stillbirth, these parents continue to memorialize their children as part of their family.

**Conclusions:**

Hospitals need to examine the physical environment for deliveries and, wherever possible, offer designated private areas with staff trained in stillbirth care. Training programs in obstetrics need to better address the bereavement needs of parents following a stillbirth, and research is needed to evaluate effective bereavement interventions, accounting for cultural variation. Critical improvements are also needed for mental health support beyond hospitalization. Finally, medical professionals and parents can play an important role in reversing the stigma that surrounds stillbirth.

## Background

"“Our daughter’s death transformed us in many ways—spiritually, emotionally, our perspective on life, and our careers as well. We are not the same people.”"

"–Father of a stillborn baby"

In the United States (U.S.), an estimated 70 stillbirths occur each day, on average 25,000 each year, with a disproportionately higher rate among African American and Native American women [1-4]. Stillbirth is one of the most devastating losses a parent can experience, and it is a high-risk indicator for post-traumatic stress and depression in mothers and in couples [5-9]. Epidemiological efforts are under way in the U.S. and internationally to better map where stillbirths occur, to better understand prevalence within and across populations [1,2,10-12]. And there are concerted efforts to increase research both to identify the causes of stillbirth and to find effective interventions to prevent stillbirths [13-16]. In the meantime, many parents in this country suffer this devastating loss, largely in silence, due to persistent stigma and taboo. While training and interventions to address neonatal and pediatric bereavement are common in U.S. hospitals, training programs or tested protocols for supporting parent bereavement after a stillbirth remain inconsistent and in need of further evaluation and development [17-24].

The guidelines for bereavement support after stillbirth that do exist require additional empirical data about what parents want or would find most meaningful and effective, with an eye to likely variation across socioeconomic and cultural contexts [25-28]. Qualitative inquiry offers opportunities to better understand the lived experience of parents against the backdrop of clinicians’ beliefs, intentions, and well-meaning efforts to support grieving parents [29-32].

The primary aim of this analysis is to offer an in-depth account of parents’ experiences of stillbirth within the context of the clinical encounter, with special attention to the emotional and personal accounts of parents, their perceptions of communication and support while in hospital and following, and their process of bereavement and attempts to give meaning to their child’s death. A secondary aim is to better understand obstetricians’ views and beliefs about stillbirth and their patients’ experiences of stillbirth, with the goal of identifying opportunities for improved communication, support, and environmental adaptations for bereaved parents of stillborn infants.

## Methods

These data were collected in the course of a needs assessment conducted by the Global Alliance to Prevent Prematurity and Stillbirth (GAPPS) to inform programmatic and training priorities for improving support for parents who lose a child to stillbirth, as well as those who have had or lost a child who was born prematurely. We subsequently realized that much of the content on stillbirths would be valuable to a broader audience and could potentially contribute to our understanding of the clinical encounter between parents and providers during and following a stillbirth. For the secondary analysis, we examined only the focus group discussions pertaining to stillbirth and conducted a thematic discourse analysis of these transcripts with attention to the how parents and physicians experienced the loss of a stillborn infant [33,34]. We present the results of 3 semi-structured focus groups conducted with parents who had experienced a stillbirth and delivered in a hospital, and 2 focus groups with obstetrician-gynecologists (OB/GYNs) from the greater Seattle region in Washington State. (Please see Table 1.)

**Table 1:** Participants and Focus Groups (FG)

**Focus Groups**	**Participants**	**Women**	**Men**
MD-FG1	OB/GYN – Private Practice N = 4	3	1
MD-FG2	OB/GYN – Academic N = 4	3	1
**Subtotal**		**6**	**2**
Parent-FG3	Mothers of Stillborn Babies N = 9	9	0
Parent-FG4	Mothers of Stillborn Babies (health professionals) N = 3	3	0
Parent-FG5	Parents of Stillborn Babies N = 8	6	2
**Subtotal**		**12**	**2**
**Total**	**N = 22**	**18**	**4**

Parent participants were identified and recruited through parent hospital guild groups and then by snowball recruitment through friends of guild members. Because the primary study was undertaken as an informal quality improvement effort, detailed demographic data (i.e., age, ethnicity, socioeconomic status, and medical history) were not collected. Despite being open to both fathers and mothers, 2 of the 3 of the parent groups consisted entirely of mothers, as reflected in Table 1. Only 2 fathers participated directly, but mothers discussed the role of husbands/partners, as did participants in the physician groups. Interviewers obtained oral informed consent from all participants. Sessions were audio-recorded and transcribed for analysis, with de-identification of the resulting transcripts. A grief counselor was present during the parent focus groups. The plan for the secondary analysis was reviewed and approved by the Seattle Children’s Research Institute institutional review board.

A qualitative progressive coding scheme was developed by the first author based on a first read through the transcripts from physician and parent focus groups as well as a literature review, and a second line-by-line read to identify central themes and concepts [35]. The initial coding scheme was condensed and the rough analysis and a rough draft were tested via member checks with two members of the physician groups and five members from the parent focus groups for input and comment [35]. That draft was revised and the second author was invited to join the analysis. The second author conducted an independent open coding of the original transcripts. Through an iterative process the authors compared and revised the coding scheme and identified value themes and subthemes within and across the conversations. Informed by the literature on parental bereavement, special attention was given to psychological impact, emotional responses, unmet emotional and social needs, and narratives of personal meaning and significance surrounding a stillbirth, especially as they coincided or differed between the parent and clinician groups. We incorporated additional peer-checking and member checking with review by colleagues in Obstetrics and Pediatrics, independent reviewers of the initial manuscript, as well as additional input from parents who participated in the focus groups.

## Results

Results are organized according to dominant discourse themes [35]. In each section, in order to preserve the discussion context within each participant group, we present supporting quotations from the parent focus groups first, followed by quotations from the physician groups [35,36]. Supporting quotations are followed by the focus group code (Table 1). When more than one participant is quoted in a particular exchange, P1-3 are assigned to indicate distinct speakers. An overarching comparison of themes across parents and physicians, keyed to major clinical practice domains, is offered as part of the discussion and summary conclusions.

### Stillbirth is an unexpected event: “You never consider stillbirth as a possibility, until it happens to you”

Stillbirth is not something a pregnant woman or couple expects or prepares for. As one mother said, “There was a ‘stillbirth’ chapter in one of my pregnancy books. I skipped it.” Many participants had believed that stillbirth is a very rare event. Several parents reported being surprised to learn the actual rates of stillbirth worldwide, or that stillbirth occurs much at all in high-income countries like the U.S. As one mother commented, “You think this is something that only happens in third world countries, but not to us with our health care resources.” (FG 5)

For all parents in this study, the shared sense was that stillbirth was something that happened to other people. Some couples in the study were pregnant for the first time and they were not considered “high risk.” Others had had other healthy children and did not expect anything to go wrong with this pregnancy. Those who were considered to have high-risk pregnancies were concerned about delivering prematurely, not about stillbirth. As one participant commented, “I was extremely high risk with all my pregnancies—and [stillbirth] was never something that came up. Nobody ever said anything.” (FG 3)

Good prenatal care was seen as providing protection against stillbirth: women who took good care of themselves and their babies would avoid problems with pregnancy and delivery. Most of the mothers in this study reported “doing everything right,” being healthy and careful.

"I had no clue. I had a textbook pregnancy and everything was perfect all the way to the end. You think that as long as you are getting medical care, they can take care of anything that is going to come up, or that there might be a warning. (FG 3)"

Stillbirth was also an unexpected clinical outcome for the OB/GYNs in the focus groups. Much like the parents who took part in these focus groups, physicians were surprised when presented with U.S. rates of stillbirth and shared the view that stillbirth primarily affects women in developing countries. When asked how well prepared they felt to address stillbirth with their patients, physicians gave mixed responses, with some feeling knowledgeable about stillbirth and others not knowledgeable (about the frequency and causes). Clinicians discussed the importance of improving education on the data surrounding the prevalence of stillbirth and causes, insofar as they may be known. Several participants remarked that they feel much better prepared to support parents through the death of a premature infant, in part because there was much that could be done to clinically manage a preterm survivor. In contrast with stillbirth, which can seemingly “just happen” with little or no warning, preterm infants who will not survive often manifest signs that enable the care team to anticipate death when it is the likely outcome.

Physicians in both groups also reported that stillbirths remain an unexpected event in their practice, since prenatal care tends to catch preventable problems early. Physicians also reported some discomfort in bringing up the topic of stillbirth with a mother or couple before it happens, often because it is typically a clinically unexpected and sudden event, but also out of concern that it may frighten the patient unnecessarily:

"We talk about it indirectly, or we talk about warning signs. Like if people are having decreased fetal movement, we tell them to come in and get evaluated. I don’t say the word “stillbirth,” but what I mean is, if things are not going well and you are not noticing the baby moving well, I want you to come in and get evaluated in the hope of picking up something early so that you potentially prevent a stillbirth, but I don’t use the actual word. It scares people. (FG 1)"

### From diagnosis to delivery, events are confusing and shocking: “No one tells you—you still have to deliver your baby”

The most unexpected reality for parents was that when a child is stillborn, a woman still has to go through labor and delivery. After receiving the devastating news that their infant has died, having to then go through the delivery experience is emotionally wrenching for parents. One woman reported requesting a Caesarean section and said the physicians told her it wasn’t allowed. Another had an emergency C-section but had no sense of what was happening or why.

Disorientation, shock, and a perceived lack of communication from the clinical team made a difficult situation harder to bear for several parents, as expressed by this mother:

"It was just so confusing. They drug you, and I think that is a huge part of it. You are on these drugs. I wake up, and first they tell me they are working on the baby. I wake up again, and they tell me that she has died. I can remember feeling a sense of disbelief, like, “No way, this is a nightmare.” (FG 3)"

Parents also described a rushed and confusing odyssey through hospital admissions, with several being admitted through the emergency room and handled by staff who were not prepared to offer information, reassurance, or support during this frightening time. Stillbirth is often confirmed by ultrasound; several parents reported encounters with technicians who were extremely uncomfortable giving or confirming the news that their baby had died.

"I had a midwife, so when I had a stress test and they couldn’t find the heartbeat, they referred me to a doctor. We had to wait through other patients before he ever came to us. He never looked us in the face. He did all the measurements. My husband finally had to say, “Is she alive?” (FG 3)"

From admission through delivery, several of the parents described feeling bewildered, in the dark, or not informed about what was happening. While partly due to the suddenness and unexpected nature of having a stillbirth, others felt that physicians purposely were not telling them why their baby had died.

"They definitely closed ranks, the doctors did. There was a shutdown on information. The nurses were fabulous, our saving grace. They encouraged us to take pictures. I had her for 24 hours and that was great, but the doctors were—it was like some kind of code of silence. You ask your doctor what happened. They say, “I don’t know.” […] There was this silence. I could just tell there was something going on that I wasn’t privy to. (FG 3)"

When physicians were asked about the time immediately following a stillbirth, most in both groups said they focused on finding answers, finding out why the baby died, precisely in order to help parents understand what had happened and why. Physicians felt torn between a responsibility to try to help parents through this difficult period, and their own sense of feeling unprepared to handle the emotional needs of patients. Many considered “giving reasons,” explaining why the stillbirth had occurred, to be their first obligation to the parents and expressed frustration with how little is understood about the wide range of potential causes behind stillbirths.

"P1: It’s obviously very, very hard for the patient, but also hard for us because we can’t answer it necessarily, why it happened."

"P2: There are some that are black and white—a cord accident, cocaine use, an abruption—and it was clear. Or, it’s a huge big gray area. There is some funky kind of clouding stuff on the placenta that maybe was there, things you look at and you are like, “I don’t know if that means anything or not.” And you are maybe grasping for an answer, but it may or may not really be the answer."

"P3: There are a lot of stillbirths that we don’t see because we just do routine prenatal care. Like we are watching blood pressures and we may induce someone before they get to the point where they abrupt. The stillbirths that I can think back on, they were just random. I mean, they just happened. (FG 1)"

Parents who experienced stillbirth wanted to understand the cause of their child’s death and found it frustrating when no answers could be given.

"Both the midwife and doctor pretty much, I feel, blew us off—“These things happen. Chances of this happening again are slim.” Then when you go home and do the research, there was testing that could have been done before I delivered. But it was testing I didn’t know about, wasn’t offered, so there could have been a cause; we don’t know the cause. An autopsy didn’t show anything; the cord was fine; the placenta was perfect. I just feel like we were blown off with, “These things happen.” (FG 3)"

The physicians in the focus groups would not routinely offer an autopsy to the parents, but would conduct one if requested. Some would offer/order lab work on the placenta if the cause of the stillbirth were not known. Several parents discussed being offered an autopsy on their stillborn baby, and a few requested that one be done to discover the cause, if possible. But this was agreed to be a sensitive subject and, as with organ donation, a very sensitive time to bring up the topic following the shock of losing the baby. Two of the parent participants who were also health care professionals requested an autopsy: “We did opt to do the autopsy after the funeral and had the funeral right there at the chapel in the hospital. Of course, they found nothing. There were no answers. Looking back on it I’m glad I did it.” (FG 4)

Physicians in both focus groups discussed the difficulty in transitioning from delivery of the stillborn to counseling or support, which was not viewed as a common role for OB/GYNs. For some physicians, the answer-giving role conflicted with the supportive role:

"The hardest part, I think, is the emotional support for us because we have to separate ourselves from that and be the clinician, and I find that is the hardest to do both—be there as their friend and then also step back and figure out why for them. (FG 1)"

Several physicians reported that they worry about being blamed for stillbirth, particularly when there is an otherwise healthy pregnancy and then the baby dies for either known (e.g., cord accident, abruption) or unknown reasons. In this case, gauging the patient and her needs feels especially critical:

"Every patient reacts differently, and you don’t know at that moment which patient this is. Is it the one that is going to blame you? Is it the one that’s going to want your comfort? And so you are caught. Probably because of malpractice, it’s like, you know, “How am I going to approach this patient?” With guarded emotions. You know? If I am close to the person, then I am comforting. (FG 1)"

Stillbirth challenges familiar roles in Obstetrics. As one participant put it, “It is hard to shift from physician to grief counselor.” (FG 1) When infants are very ill and at risk of death from conditions related to prematurity or other congenital conditions, they are typically handed off to neonatologists or pediatricians. These deaths occur in neonatal intensive care units (NICUs), outside the ambit of obstetrical care.

"[With stillbirth] you transition from being a doctor to a hand holder, a grief counselor. Compared to prematurity—you know you have a baby; you’ve got a premature baby. You become hopeful, kind of a cheerleader along the way. The team transitions too, because as an obstetrician once pregnancy ends, while you are still involved, that baby gets taken over by the pediatrician or pediatric team. I won’t say the relationship ends, but it definitely changes. Whereas with stillbirths there is no transition of care, and you are kind of left being that counselor. (FG 2)"

Because infant death is much more common on neonatal units, clinicians working on NICU teams are likely better prepared to implement palliative care practices. Indeed, the grief kits and photography described in the parent focus groups are approaches that were initially introduced in the neonatal context. Several of the obstetricians in our groups reported feeling underprepared for a shift to palliative care and counseling and tended to defer to social workers or nurses to provide needed support for patients, or to other families who have experienced stillbirth. 

### The typical labor and delivery environment is not designed to support parents during a stillbirth: “I could hear other babies crying”

Delivery areas on obstetric units are designed to welcome new babies, not to mourn babies who have died. Parents and physicians alike described challenges related to the set-up and structure of the typical labor and delivery floor. Delivery rooms are not typically set up to provide space or privacy for parents who have lost a child before or during delivery, and the culture and milieu of labor and delivery is to give birth. The birth of a baby is associated with bustle and noise—the baby’s crying, the flurry of activity after birth, the congratulations and cheers, the phone calls to share the happy news. By contrast, stillbirth ushers in silence. As one mother described the poignant experience of holding her stillborn baby:

"When I first held him, he was warm and soft and wet like a newborn should be. But he was silent. I held him as his body grew cold. I tried to keep him warm, but I couldn’t. (FG 5) "

Several mothers reported that hearing noises of the bustling activity and other births around them added to their suffering.

"They left me in the same floor as everybody else. You hear babies crying. I mean, that was horrible. They put a sticker on your door. I was like, “What is that for?” They’re like, “Well, it’s to let everybody know that you don’t have a baby here.” It was just horrible. (FG 3) "

The default assumption that all parents in the labor and delivery suite are celebrating the joyous occasion of a healthy birth can be devastating for parents who have a stillbirth, and physicians in the discussions appreciated this tension and described efforts to offer privacy. One parent reported being transferred to the geriatric ward where the staff thought she would have more quiet and privacy—a well-meaning attempt to address the lack of a private space on OB. However, the result was that the ward staff wondered what she was doing there, and the patient had to explain, several times, that she had been moved out of OB because she had had a stillbirth. Three other mothers shared their experiences with clinical staff following a stillbirth:

"They didn’t put a sticker on my door, so they kept coming in—“Do you have your baby?” That was horrible because they came in two or three times. They came in to take pictures of him, thinking he was alive. I don’t think they knew what to do—it was horrible. (FG 3)"

"The nurse kept coming by and saying, “What are you hoping for, a girl or a boy?” So they weren’t even reading my chart about the baby having already died. Then I was in a room with all of the women delivering babies, because it was a smaller hospital. (FG 3)"

"The stupid lullaby that plays every time a baby is born—we could still hear all of that. We had a private room, but we could hear the chirp lullaby chiming away all the time. (FG 3)"

Both physicians and parents described the painful ritual of parents leaving the hospital or delivery suite without a baby. Shifting from the joyous expectations of a new baby with waiting baby rooms, expectant friends and family, and to grief and loss is not something any expectant parent is prepared for: “We were planning to bring home a baby and had to immediately start planning a funeral.” (FG 5)

"One of the hardest things ever was walking out of the hospital empty handed. Somebody had brought me a little potted flower basket and I carried that out and I was so glad I had just that. It’s just that feeling of walking out empty handed. Of course, what I really wanted was a baby to walk out with. (FG 3)"

### Clinician empathy and rituals to honor a baby’s death are important to parents: “They encouraged us to take pictures—I’m glad we did”

All but two of parents in the focus groups expressed frustration about how clinicians and staff handled their delivery of a stillborn as described above, but it is worth reporting those gestures that two parents found to be meaningful and supportive, offering an insight into what parents find comforting:

"I had a wonderful doctor. I felt completely taken care of and I was sent home with a huge packet of information. Stuff was mailed to me that the hospital had set up. A pastor came and dedicated him. A social worker came in and talked to us and told us kind of what was going to happen to us as a human in grief. (FG 3)"

"It was wonderful as far as the staff. Somebody told us that we would appreciate having pictures. It would never occur to me to take pictures of him. We took lots of pictures and I held him for about 3 days. At different times they would bring him in and I held him. The last day I put a diaper on him and dressed him in a nice little outfit. He was buried. I just thought they were wonderful. (FG 3)"

Other positive accounts of the hospital experience included thoughtful gestures of compassion and support, such as giving parents time to hold their baby, offering to photograph their child, and offering to bathe and dress the child. For one couple, the staff assumed that the mother needed rest and asked the father whether he wanted to bathe and dress the infant. The mother mentioned bathing and dressing as very positive gestures but felt left out of an opportunity to create memories with her child, underscoring the importance of considering the needs of both parents, as a couple and as individuals: “I think they were concerned with me resting. My husband went with them to dress her. He got to bathe her but it was never offered to me to bathe her.” (FG 3)

Even for parents who wanted and were given time to hold their dead child, the immediate aftermath of delivery was jarring and shocking, after the otherwise healthy pregnancy and preparation for the birth. Not all parents found providers’ well-intentioned gestures, or their timing, comforting. Others appreciated the gestures only much later:

"After my daughter was born and they told me she was dead, I didn’t want to see her. I didn’t want to see what a dead baby looked like. They basically, for lack of a better word, forced me to do it. I had nurses in there. I was hysterical. They said, “You have to hold her and say goodbye to her while she is still warm. Don’t wait for her to be put in the refrigerator. Don’t go on 10 years and look back and say you never held her, you never saw her.” I was so angry with them. I was yelling, but when I look back, that was the crucial event that they did to not only preserve future memories but for me to get through it (…) They were right. (FG 4)"

"The tech offered to have me look at the ultrasound. I hit his hand away. Why do I want to look at a picture of him on the ultrasound—so I don’t see the blood flowing and don’t see the heart beating? It was very harsh. I think maybe if we are treated more like shock patients. You go into a state of shock. (FG 3)"

Most meaningful for all parents were the physicians and nurses who took time to sit with them, look them in the eye, and be present with them in their sadness. One of the mothers who reported an otherwise terrible experience at the hospital remarked that what meant the most was a nurse who sat with her and told her she had lost a baby too: “She was personal with us, so we actually go and visit with her every year on the anniversary of our daughter’s death.” (FG 3)

All parents valued eye contact, empathy, and emotional engagement from their physicians. One mother was very moved by her physician’s grief.

"P1: My doctor was very compassionate and looked me in the face and said, “I’m sorry, your baby has died.” I’ve heard a lot of stories about physicians that didn’t look their patients in the eye. I think that is something that was important to me."

"P2: I was told my doctor cried. He stepped out of the room and cried."

"M: How did that make you feel?"

"P2: I just felt he was very compassionate. (FG 3) "

Here, where this physician’s instincts or training may have led him to attempt to protect the patient from his emotional reaction, the patient instead found the reaction to be deeply human and a sign of shared grief over a terrible loss.

Parents who were offered to have a photograph of their baby taken appreciated this and said it was not something they would have thought of, but appreciated later. Others had handprints or footprints taken. One hospital offered a loss kit that included a blanket, footprint, handprint, and a camera. Other parents had friends or families offer such gestures to mark and remember the birth of their baby:

"P1: We had girlfriends that came and got a mold of her hand and a handprint and stuff like that. The hospital didn’t have that resource but could have said, “Hey, you might want to…”"

"P2: Did they do a footprint?"

"P1: They did, but just on a piece of paper."

"P2: Because later I wished I had had the hand. Sometimes I don’t remember her hands. I didn’t see her hands so I have those things that I can go back to. (FG 3)"

### Grief following stillbirth is ambiguous: “My saddest memories are also the ones I cherish”

All of the mothers described significant grief and depression for weeks and months following the loss of their child. Several were offered anti-depressants in the hospital. Information and materials were typically offered by nurses or social workers. Only a few mothers in these groups were given referrals for counseling; most sought counseling or treatment on their own. Most had family members or friends stay with them at home in the weeks after the death of their baby. One father described the grieving process as uncertain and changing over time:

"The grieving process was very ambiguous for me and my wife—we didn’t know what to expect, it took a while to process, and trying to understand what to do became a daily/weekly challenge. (FG 5)"

Both of the fathers who participated in these discussions described feeling sadness or depression, but supporting their wives was their primary focus. This was echoed by the women in the groups; when asked about their husband’s grief following loss of their child, they shared their sense that men are deeply affected by the loss but process the grief differently, focusing on helping their wives through the grief, and trying to find solutions for recovery.

"P1: My husband tried to be strong for me."

"P2: My husband just wanted to “fix it.” (FG 5)"

When asked about his own response, one of the fathers described mixed emotions during the months following the loss of their child, including anger toward those who didn’t seem to understand the depth of their grief over a stillbirth: “Yes, depression, but more anger—anger at the medical system, and friends, family, and ourselves.” (FG 5)

All of the parents felt largely on their own in identifying options for grief support and expressed a desire for more information and better resources. Parents had different needs: some wished for immediate social support, while others felt unable to talk about their experience for some time.

"P1: I was desperate to go to a support group. I read that booklet [When Hello Means Goodbye] over and over again. I was desperate for information. I got a computer and started going onto boards. There was a stillbirth board. I called everybody I knew to see if there was anybody that could help me. Every day was such a struggle. It was really tough. So other people helped enormously."

"P2: We did venture out to a support group, which felt like a huge risk at the time. I felt like I couldn’t get there fast enough every month. Those are the people I have become closest to. The relationships were the most important thing."

"P3: I didn’t want to go anywhere or do anything, so I don’t think I would have wanted to go to a group and talk just because I was so depressed. (FG 3)"

Parents mentioned the value of social work referrals, information on counseling and parent support groups, as well as simple human compassion for their loss. Several women mentioned the value of talking with someone who had gone through this before. They discussed the idea of creating a mentorship program, making themselves available as guides for other women who experience stillbirth, both as something they wished they had had at the time of losing their child, and as a way of helping them find meaning and value in their own experience—being able to then help other parents.

Parents in these groups very much want to keep their child’s memory alive, and to hold on to the few memories they had of their child at birth—holding their baby, the shape of hands and feet, the look of their face—and yet they do so knowing that these memories also will trigger sadness. As one mother said, “My saddest memories are also the ones I cherish.” (FG 5)

### What to say and not to say following a stillbirth: “Don’t say, ‘You are young—you can have another’”

Many of the parents’ comments focused on well-meaning but hurtful comments from clinicians who meant to be supportive but simply did not know what to say or do to offer comfort after a stillbirth. For parents in these focus groups, the most common and most hurtful comments were reassurances that they would have another baby.

When asked what clinicians should say to parents, one mother responded, “Don’t say, ‘You are young—you can have another.’” (FG 5) Parents experienced variations on this statement as dismissive, failing to recognize or respect what they had been through. For others, poor timing was an issue: “In the midst of grief over the loss of your child, you’re just not thinking about your next pregnancy.” (FG 5)

"P1: I think if your doctor is going to focus on anything, he is going to focus on the baby that lives. You’ve had a stillbirth. There is nothing they can do about it, and they are like, “that’s it, go on.” How many of us heard “You can have another baby”? Go have a baby. Like that is going to solve everything, if you have another baby."

"P2: Other people say that. My dentist said that."

"P1: It is beside the point to have another. I wanted that baby. (FG 3)"

Overwhelmingly, mothers were deeply upset by comments that physicians intended to be reassuring—instead, such comments made the mothers feel as though the physicians did not appreciate that they had just lost a particular, loved baby, and that for a parent, there is no substitute for a dead child.

Several of the women said the biggest lesson following a stillbirth was “to trust your instincts about your body.” (FG 5)

"There are so many women I talk to after their loss that said “I kind of thought something was wrong, but I didn’t want to go in.” They talk themselves out of it, and so I think that would be nice to know ahead—for me, just so I wouldn’t have talked myself out of going to the doctor. (FG 3)"

As one mother who had experienced stillbirth reported, she now tells other pregnant women to count the kicks and movements toward the end of pregnancy and record them to make it easier to share with the nurse or physician. (FG 4) Parents felt that more training needed to be done to educate clinicians for supporting parents, especially the women, who had a stillbirth and then went on to become pregnant again. With the next pregnancy, there can be intense feelings of fear and anxiety. While a prior stillbirth may not be sufficient to medically qualify a woman as high risk if all else in the pregnancy is normal, women who went on to have another child after their stillbirth said that they had felt extremely anxious about their subsequent pregnancies. They reported wanting added precautions, monitoring, and attention to their pregnancy: “I was not viewed as ‘high risk’, so I couldn’t get into the high-risk pregnancy service, even though I tried.” (FG5) Others described wanting an earlier C-section toward the end of their pregnancy: “I wanted her out! I wanted her while she was still alive.” (FG5)

All of the physicians acknowledged that they struggle with what to say and not say following a stillbirth. Some felt that we simply do not understand why many stillbirths happen—aside from those that were due to a clear cause, such as abruption. Others felt that the topic was not well covered in their training, and that providing more information on prevalence and causes in training and practice would help to prepare physicians for these conversations. For most of these physicians, communicating with parents following a stillbirth was seen as a challenging task. Several of the physicians were less inclined to encourage mothers or parents to talk about their feelings unless initiated by the patient, out of concern that they may make parents feel worse.

Most physicians thought obstetricians should be equipped to provide parents with medical knowledge, information on why the stillbirth may have happened, if information could be gained through further testing. As noted earlier, many physicians saw giving reasons as their primary responsibility to parents following stillbirth and felt frustrated when they could not offer clear medical reasons to the parents.

"P1: It’s hard. Sometimes once you have done the workup and told them you can’t find any reason, or this is the reason and this what you are going to need next time, I think sometimes you still can’t give them everything they want. Sometimes they just need to move on. (FG 1)"

"P2: You try to figure out why it happened. And prepare them for the future, depending on what the result was, prepare them for a future pregnancy if they decide they want to pursue another pregnancy. (FG 1) "

Where reasons could not be found, physicians tended to focus on the future, on encouraging a woman to move forward, and on reassuring the parents that in all likelihood they could go on to have another healthy pregnancy. This last point was importantly couched in terms of easing a woman’s mind and countering a woman’s potential feelings of self-blame.

### Parents struggle with the silence and taboo that surrounds stillbirth: “I know it makes some people uncomfortable, but I want to talk about my daughter”

All parents in the focus group discussions described a range of emotions surrounding the loss of their baby. They described feeling shell-shocked, devastated, overwhelmed, numb, angry, grief-stricken, empty. They described all the emotions of loss and grief that would attend the death of any child or loved one, but without the social space to legitimize it or make space for expressing those emotions.

"As a society we really haven’t given it a place, so if somebody did have that experience it was very quiet… [T]here isn’t an appropriate way to grieve a child that is stillborn, especially when people have never seen it. The only people I have met that have their own ways of grieving and celebrating are in support groups or around a community of people that have experienced the same thing. Outside of that community there is just this void, nothing. (FG 3)"

The silence and loneliness that these parents describe, long after the death of their baby, are palpable. Much of the isolation is caused by the awkwardness and discomfort felt by others when parents of a stillborn try to discuss their experience, or when they try to normalize it by mentioning their stillborn child alongside their live children as part of their family.

"When people ask me, do you have kids, or how many, I have to check myself. It depends who I’m talking to, and whether they get it. But if it’s people comfortable with this, I say, I have three kids—two living, and a daughter who died, [gives daughter’s name]. (FG 5)"

Social taboo, stigma, and silence were experienced as significant sources of distress for parents of a stillborn child—not being able to grieve openly, and not being able to openly celebrate or remember their baby’s birth and death, long after the experience.

"P1: We like to hear our child’s name out loud."

"P2: I know it makes some people uncomfortable, but I want to talk about my daughter. "

"P3: To me, whether my child was stillborn or died right after birth makes no difference to me—only to you. (FG 5)"

Next to the silence or taboo that surrounds stillbirth, parents of stillborn children identified the lack of understanding or support from family and friends as one of the most difficult parts of the experience and its aftermath. Parents were asked to write down 2 or 3 things that they wish care providers, friends, and family would understand about their experience and what type of support was helpful or hurtful. These are summarized in the list below.

I am still a mother/father, even though my child died.

A child was BORN.*

Losing a baby who lived outside the womb is not *worse* than losing a baby before birth.

The love you feel when you have a dead baby isn't different than a living one. We LOVE our child(ren).*

Dads miss their child, too.

It’s okay to talk about it. You can’t make me more sad.

Don’t worry about making me cry when you ask about my child; I cry anyway.

The people who said nothing were worse than those who said something.

Someone said to me, “It’s a good thing you didn’t bond with your baby.”

We are not “lucky” that our children died before we brought them home.

Pictures of our dead child might be beautiful to us.

We are not mentally unstable because we like to honor/celebrate/remember our stillborn child.

Stillborn children have funerals too.

My baby is (and will always be) an important part of my life and family.

I’ve held my dead child, and I long for those moments.

We will never be “over it.”

No child will replace our lost daughter.

(*Emphasis added by parents.)

Perhaps the strongest theme across the parent group discussions, and the sentiment parents most wanted to convey to others, was that their stillborn child is real and will always be remembered as part of their family. The parents in these groups named their children, and several discussed the importance of having a record of the birth, and at the very least a death certificate. While they identified strongly as parents, and said they will always be parents of their stillborn child, they felt that this part of their identity is not recognized by others.

"I am a mom but you don’t get that recognition. (…) Just the birth certificate—I was really fixated. I want a piece of paper to say that my child existed. (FG 4)"

The parents describe the grief of stillbirth as being just as deep, painful, and significant as it would be to lose an infant who is born and survives a few weeks in intensive care. However, other people—including, according to parents in this study, some health care providers—do not treat these deaths as equivalent.

"[T]o this day, there are people who get this uncomfortable look on their face. The grief is always in your heart. You carry it every day. Then when you get some reactions from people—they are so uncomfortable—it just makes it hurt even worse because you can’t talk about your two sons because only one is the acceptable one to talk about. (FG 4)"

Beyond the hospital, parents also shared the challenge of returning to work and going about routine social events while still deeply grieving the loss of their child, when those around them did not perceive the loss as equivalent to the loss of a preterm infant, or another child who lived some time before dying. This sense of constrained grieving caused by social discomfort and taboo extended to husbands and grandparents, who were not expected to grieve the loss of a stillborn baby beyond feeling some transient disappointment or sadness for their wife or daughter.

"People would call my husband and only ask how I was doing. What about him? (FG 5)"

"My parents, they lost their first grandchild. No one comforted them or asked how they were doing. (FG 5)"

"My husband, the role that he associates most with is—because I carried the babies, but he put them in the ground, and that is what he remembers doing—burying the babies. He made it his point to take their little casket and put them in the ground. That to me is just as emotional and just as painful as the physical pain that I went through. (FG 4)"

Parents noted that the entire family—including siblings and grandparents—is affected by a stillbirth. Aside from funerals, they described beautiful ceremonies and ways of remembering their children as a family. All parents interviewed mark the birthdays of their child in various ways. Many have a designated place of remembrance, such as the burial site, a commemorative bench, or an engraved stone in the garden. Others take family trips every year to mark the birthday of their lost child, and described this as a time of family celebration. Some imagine what their child would be doing had they survived. One mother described keeping a journal for her daughter. She described writing to her, imagining what age she would be, and shares the events of her day and the hopes she had for her daughter. In a social context that does not readily embrace practices or rituals to honor the death of a stillborn child, these parents describe ways of quietly creating rituals of great meaning and significance to them and their immediate family.

All of the obstetricians in the groups acknowledged the social taboo surrounding stillbirth and thought it an important role for physicians to try to counter any self-blame on the part of parents, in those cases of stillbirth where the cause was not due to known, risky behavior.

"P1: They usually blame themselves; they think it’s their fault. It’s our role to make sure this is not happening and reassuring them that there is nothing they did. It’s very important to reassure them that they did not do anything. (FG 1)"

"P2: It’s not the mom’s fault. I mean, except where you know the cause is something like cocaine and they knew not to do that. The flipside is, you don’t want everyone walking around in bubble wrap during their entire pregnancy. (FG 1)"

Two physicians discussed strategies for neutralizing patients’ perceived self-blame by “normalizing the experience,” with phrases like “this sometimes just happens,” and by explaining how surprisingly common stillbirth is, even in very high-resource settings and excellent hospitals, and even when a mother does everything right during a pregnancy. Being more informed about the rate of stillbirth and its causes, both groups of physicians agreed, could help obstetricians provide meaningful context for parents, to let them know they are not the only parents this has happened to. 

## Discussion

Juxtaposing the experiences of parents and physicians surrounding stillbirth can help elucidate areas of common understanding and areas of potential misunderstanding between families’ and clinicians’ respective feelings and perceptions during this very emotional and challenging clinical encounter. (Please see Table 2) Parents’ stories about the hospital experience included both positive reports of supportive providers and a private setting, as well as wrenching accounts of unprepared providers and the added devastation of being surrounded by the sights and sounds of healthy deliveries. A few parents and clinicians mentioned the use of grief kits and other programs to help parents bring meaning to their child’s death, which have been adopted from neonatal and pediatric palliative care practices [19-23]. However, based on the feedback from this group of parents, there is still a gap in training and variations in supportive practices across hospitals and practices that may need to be more explicitly extended and evaluated for Obstetrics training programs and practice.

**Table 2:** Comparison of Parents’ and Physicians’ Beliefs and Feelings

	**Practice Domain**	**Themes: Mothers/Parents**	**Themes: Physicians**
**1**	**Knowledge/awareness**	Believe that stillbirth happens more in low-resource countries.	Believe that stillbirth happens more in low-resource countries.
		Never expected this to happen and were shocked and unprepared.	NICUs are better prepared to handle deaths.
		Wanted to know the reason but no one could tell them the reason.	Except for obvious causes (cord/abruption), doctors often don’t know why stillbirths happen.
**2**	**Hospital – environment**	Being surrounded by reminders and sounds of healthy deliveries causes feelings of humiliation and anger on top of grief.	Believe that efforts are made to offer privacy and sensitive support.
		Overwhelmed, adding insult to injury, by having to explain (sometimes repeatedly) what happened to unprepared staff.	Accustomed to handing off patients (mother and infant) to a pediatrician or neonatologist.
		Appreciated the presence of physicians, social workers, and nurses who provided support to parents in grief and bereavement.	Most feeling unprepared to shift from role of physician to counselor.
**3**	**Hospital – care & communication**	Wanting physicians to engage them in their sadness and grief.	Wanting to suppress/hide sadness and grief to focus on finding out ‘why’ for the patient.
		Feeling shut out, not knowing.	Wanting to figure out the cause through tests or autopsy.
		Wanting answers but not strongly blaming self.	Wanting to reassure mothers in particular not to blame themselves.
		Holding someone responsible: Some blame the hospital or physician for not preventing the stillbirth.	Some worried about being blamed.
**4**	**Rituals around death**	Holding, bathing, dressing, photos, hand and footprints are important and preserve memories. However, should be offered, not forced.	Some offered death kits similar to those used in NICUs; others felt unfamiliar with what a parent might want and would defer to nursing staff.
**5**	**Post-stillbirth care**	Described grief as ambiguous—wanting to remember deeply sad memories.	Focused on reassurances about the future. Referrals to social work but few referrals to mental health professionals for follow-up care.
		Hunger for information on causes, prevention, and support resources. Few were offered this in hospital.	Typically make referrals to nursing or social work to offer information.
		Parent support groups and online groups helpful—someone who has gone through stillbirth and come out on the other side.	Not aware of these resources.
**6**	**Post-stillbirth communication**	This baby mattered to us and another baby will not replace her or make our feelings go away.	It is important to reassure mothers they did nothing wrong and can have another baby.
**7**	**How stillbirth is perceived**	A stillbirth is the death of a child.	A stillbirth is not as severe a loss as the death of a neonate.
**8**	**Bereavement, remembrance, & recovery**	We want to openly remember our baby as part of our family.	It is important to try to help them realize they have done nothing wrong and can go on to have another child.

A more standardized approach has been adopted throughout maternity units in the U.K. This includes offering photographs and footprints, and strongly encouraging parents to hold their stillborn infant. One important but small study raised doubts about the effectiveness of such approaches in helping parents recover; results suggested that holding or even seeing one’s stillborn baby was associated with higher rates of long-term depression and post-traumatic stress [25]. However, in addition to the serious limitation of not reporting why parents did or did not choose to hold their baby, this study is limited in not considering the value parents may place on creating emotional ties with their baby despite the potential psychological costs of doing so (potentially longer depression or grief). The majority of parents in our study said that they want to grieve their children and do not want to forget, even if this causes or prolongs the pain of loss. Several described this pain as part of them, as something they would always live with. The comments about wanting to remember what their baby looked like, their hands and feet, demonstrates another value for parents who have lost a child: memories. Even if those memories prolong or reignite later grief, some parents may find that pain preferable to living with the irremediable regret that they never saw or held their baby. This study underscores the need to carefully evaluate our palliative care and bereavement approaches and for individual clinicians to sensitively gauge parents’ needs, rather than a one-size-fits-all approach. This is supported by what we do know about variability among people and across cultures when it comes to the phases of grief and recovery [24,29,36-38].

These parents also reinforced what we already know about the value of clinician empathy, direct eye contact, and sympathetic engagement [39]. Several of the parents witnessed their physicians showing signs of sadness at their loss, and these signs were quickly hidden, as in the instance of the physician going out in the hall to cry. These may be difficult but powerful opportunities for a physician and patient to connect in grief, and to help each other through a tragic human experience. Both physicians and parents were together in wanting answers, wanting to know why the stillbirth happened. However, timing is critical. Some physicians were trying to gather answers and not wanting to talk to parents until they had information to offer. Some patients were feeling left out of this process, and wanting emotional engagement immediately following the loss of their baby. Given that the future-oriented comments from physicians that were meant to be reassuring were often felt by parents and mothers to be unintentionally hurtful, the best initial approach may be non-verbal—perhaps holding a patient’s hand or simply sitting quietly with the patient in what one group of experts in cancer communication has termed “compassionate silence” [40]. There will also be value in helping parents to connect with peer groups where they can feel supported and understood, particularly during the immediate grieving process, but also for long-term, ongoing support in a social context that still does not make much room for grieving or remembering a stillborn child [17,32,41-43].

Our data also reinforce what has been reported elsewhere regarding the ambiguous nature of grief surrounding stillbirth, from both the parents’ perspective as well as the social or cultural perspective [44-46]. The women and couples who experienced stillbirth are, in their experience, grieving parents, and yet several parents in our groups reported that physicians as well as friends and family see them as something else—perhaps as people who have undergone a terrible loss, but not as parents who have lost a child. While physicians recognized the loss, they did not seem to grasp the depth or significance of the loss to the parents. For physicians, “it was *like* [our emphasis] losing a family member.” For parents, it *was* losing a family member. These parents of stillborns were emphatic that they wanted to talk about their children, and that they very much view their babies as any other baby who took a first breath. Their hope is that we reach a point where bereavement and remembrance can be more openly expressed and embraced and understood by others.

The parents in this study are voicing a more widely known phenomenon that most women and many parents do in fact bond with their baby over the 8 to 9 months of pregnancy [27]. And yet, socially and even medically, this bond is not appreciated. Greater appreciation of such attachment can help clinicians recognize symptoms of depression associated with stillbirth, long after hospitalization [42,43]. These stillborn infants count to these parents, and the love and sorrow the parents feel are real. For these parents, failing to recognize their grief or to offer supportive space for continued remembrance of their lost children is robbing them of their identity as parents, based on the fact that their baby did not survive birth.

On the spectrum from very early miscarriage to a neonatal death, the physicians in this study conceptualized stillbirth as more like a miscarriage than like the death of an infant, whereas parents see it the other way around. The metaphor of taking that first breath in the world carries strong moral significance for many people even though, at the end of gestational development, such a cutoff makes little sense medically and is essentially arbitrary [47]. Terms like fetal demise or perinatal loss, while commonly used in clinical settings, invoke euphemisms for death. For many of these mothers, the term “stillbirth” captured a number of important realities: in stillbirth there is a birth, somebody was born, and someone did the birthing. We can then better appreciate how using euphemisms may unintentionally minimize the experience from the parents’ perspectives.

The majority of physicians in our groups perceived their role as clinicians or scientists to be bound up in finding answers for the parents, and some found it challenging to focus simultaneously on the clinical situation and the emotional needs of the patient in a charged and often clinically complex situation. Further, physicians who believed that medical knowledge or expertise is what they are best able to offer the patient, and ultimately the cause of the stillbirth could not be determined, felt frustration in not having something to offer the parents [48]. One communication model from oncology demonstrates that patients at the end of life value the physician’s ability to move fluidly between providing recognition of suffering, emotional support, and guidance based on clinical expertise [39,40]. While parents did want answers and information about what was happening during the bewildering medical odyssey of delivering a stillborn baby, they also wanted and deeply appreciated simple human kindness and empathy from their physicians and nurses.

Clinicians importantly emphasized the goal of reassuring the parents that they had done nothing wrong and that they could likely go on to have another healthy pregnancy. This is an important insight given that mothers did want to understand why this happened, despite their efforts to “do everything right.” However, the timing of attempts to encourage future-oriented thoughts, and shifting attention to the next pregnancy, had the opposite effect intended by physicians. Parents said they wanted to openly grieve their child. After grieving their child, they wanted to create memories around their child and not forget them. “Moving forward,” for parents, was finding a place for the grief and making a place for remembrance and celebration of their lost child [49]. This misunderstanding among our groups suggests that physicians could incorporate conversations about memorializing the stillborn baby as an important part of helping parents cope with the loss rather than emphasizing the next pregnancy, since this was seen as discounting the worth of the baby who died.

There were a number of limitations to our analysis, and these can help inform and strengthen future studies on this topic. The results reported are from a secondary analysis of focus group data that were generated in the course of an informal needs assessment to inform a new program focused on prematurity and stillbirth. As such, there are a number of unavoidable limitations due to not having a hand in study design. Important demographic data such as age, socioeconomic and ethnic background, and time since stillbirth, were not gathered and are not available. A conservative assumption in generalizing from our data should be to assume that the majority of parent participants likely reflected the demographics of the Seattle area hospitals represented by the parent guilds used in recruitment: middle-income and higher-income families, and predominantly Caucasian. For this reason, the sample certainly fails to represent all of the diverse cultural and socioeconomic views surrounding this complex topic [31]. Additional work is needed to better understand variations in cultural beliefs and experiences of stillbirth in the U.S. context, as well as additional socioeconomic barriers to care and support from participants of greater socioeconomic diversity. In addition, the questions posed were very open ended and exploratory, which offered the advantage of openly eliciting salient themes regarding stillbirth bereavement, but did not include the kind of targeted probes and follow-up questions that would have been part of a carefully designed qualitative study based on existing literature. The data also over-represent the views of mothers. We still have very little data on fathers’ experiences of stillbirth, and while fathers were invited to participate in these discussions, very few did so [50]. To correct for this limitation, mothers were asked about their partners’ feelings and experience, and they often shared both perspectives. The fathers who did participate said they wished more fathers would talk about losing a child to stillbirth. However, they also felt that their primary role was to support their partners, and so saw their grief or needs as taking a back seat. These are sentiments worth exploring in future studies. Despite these limitations, the dominant themes that emerged from this analysis, including the depth of the parents’ grief surrounding the loss, and the lack of social support or empathy during the grieving process, echo themes found in the emerging U.S. and international data on the experience of stillbirth and confirm their broader validity [27,29,31,32].

## Conclusions

When we consider the major themes of parent and physician discussions side-by-side and against the backdrop of clinical practice domains, we can identify opportunities for improving communication and support for parents of stillborn children throughout and following hospitalization. (Table 2) Our findings confirm a shared theme in the literature on the psychological impact of stillbirth on parents: the grief and devastation are deeply felt but not socially recognized [7,24,27-30]. This is true especially in the months and years following a stillbirth, when parents continue to mark birthdays, memorialize their children, and will always consider them a lost member of their families.

There are several important practical implications of our findings: First, medical schools and training programs in obstetrics, as well as continuing education programs for established clinicians and staff, could benefit from more targeted training on the bereavement needs of patients and their spouses following a stillbirth. Critical improvements are needed for mental health support and counseling beyond the brief period of hospitalization [42,43]. And research is needed to evaluate particular interventions and practices that take into account cultural variation among patient populations [31]. Second, hospitals need to examine the physical environment for deliveries and, wherever possible, offer designated private areas with staff trained in patient care following a stillbirth. Third, physicians and medical professionals can play an important role in reversing stigma and social attitudes surrounding stillbirth, first in communicating directly with their patients but also by speaking out on this issue in published work or in the training of future clinicians. Finally, parents of stillborn infants can play a critical role in helping clinicians better understand what supportive interventions are most helpful following a stillbirth, as clinicians, too, struggle with how best to support patients during this sad and tragic experience.

## Competing interests

The authors have no competing interests to declare.

## Authors’ contributions

MK conducted the secondary qualitative analysis of the transcripts and wrote the initial draft. MK revised the initial draft based on input from ST and a subset of participants and physician colleagues. ST and MK conducted a joint second review of the coding and thematic analysis. MK and ST wrote the final draft. Both authors read and approved the final manuscript.

## Pre-publication history

The pre-publication history for this paper can be accessed here:

http://www.biomedcentral.com/1471-2393/12/137/prepub
